# Immunotherapy for Metastatic Triple Negative Breast Cancer: Current Paradigm and Future Approaches

**DOI:** 10.1007/s11864-023-01069-0

**Published:** 2023-04-20

**Authors:** Veerle Geurts, Marleen Kok

**Affiliations:** 1 Division of Tumor Biology & Immunology, Netherlands Cancer Institute, Plesmanlaan 121, 1066 CX Amsterdam, The Netherlands; 2 Department of Medical Oncology, Netherlands Cancer Institute, Plesmanlaan 121, 1066 CX Amsterdam, The Netherlands

**Keywords:** Breast cancer, TNBC, TILs, Immunotherapy, Immune modulatory strategies

## Abstract

In approximately 15–20% of the patients diagnosed with breast cancer, it comprises the triple negative (TN) subtype, which until recently lacked targets for specific treatments and is known for its aggressive clinical behavior in patients with metastatic disease. TNBC is considered the most immunogenic breast cancer subtype due to higher levels of tumor infiltrating lymphocytes (TILs), tumor mutational burden and PD-L1 expression, providing a rationale for immunotherapy. The addition of pembrolizumab to chemotherapy as first-line treatment resulted in significantly improved PFS and OS for PD-L1 positive mTNBC, leading to FDA approval. However, response rate of ICB in unselected patients is low. Ongoing (pre)clinical trials aim to further optimize ICB efficacy and widen its application beyond PD-L1 positive breast tumors. Novel immunomodulatory approaches to induce a more inflamed tumor microenvironment include dual checkpoint blockade, bispecific antibodies, immunocytokines, adoptive cell therapies, oncolytic viruses, and cancer vaccines. Preclinical data for these novel strategies seems promising, but solid clinical data to further support its application for mTNBC is awaited. Biomarkers capturing the degree of immunogenicity such as but not limited to TILs, CD8 T cell levels, and IFNg signatures could support deciding which therapeutic strategy is most appropriate for which patient. Given 1) the accumulating therapy options for patients with metastatic disease and 2) the heterogeneity of mTNBC from inflamed to immune-desert tumors, the challenge is to work towards immunomodulatory strategies for specific subgroups of patients with TNBC to enable personalized (immuno)therapy for patients with metastatic disease.

## Introduction

Breast cancer is the most commonly diagnosed malignancy among women worldwide. In approximately 15–20% of the patients, it comprises the triple negative (TN) subtype defined as lack of expression of the estrogen- (ER) and progesterone receptor, and absence of human epidermal receptor-2 (HER2) overexpression or amplification [1, 2]. Patients with TNBC are at higher risk for an early recurrence, with the majority of recurrences occurring in the first 3 years after diagnosis [3, 4]. Around 30% of the patients with early-stage TNBC will develop distant metastases, whereas 6–10% of all TNBC patients present with de novo metastatic disease [5, 6]. The predominant metastatic sites in TNBC are lymph nodes and visceral organs. Advanced TNBC has a poor prognosis with a median overall survival of only 8–13 months [7, 8], emphasizing the need for novel treatment options to further improve survival of mTNBC-patients. This review focusses on recent developments in the field of immunotherapy, the current challenges and novel immunomodulatory avenues to improve treatment of mTNBC.

## Immunological properties of mTNBC

Historically, breast cancer is considered a ‘cold’ tumor type characterized by a less inflamed tumor microenvironment (TME) as compared to ‘hot’ tumors with high levels tumor infiltrating lymphocytes (TILs). In-depth characterization of breast tumors revealed strong heterogeneity among breast cancer subtypes, with TNBC showing higher levels of TILs, PD-L1 expression and a higher tumor mutational burden (TMB) [9, 10]. These key characteristics are indicative of pre-existing immunity and have been associated with response to immune checkpoint blockade (ICB) in other tumor types, and more recently with outcome after anti-PD1 in mTNBC [11, 12]. Therefore, TNBC is considered the most immunogenic breast cancer subtype.

Immunogenicity diminishes towards more advanced disease stages with metastatic breast cancer (mBC) being more immune depleted as compared to primary tumors illustrated by a decrease in TILs, lower PD-L1 expression and lower likelihood of response to ICB [12–14]. The amount of TILs even varies between metastatic sites [13]. A shift towards more immunosuppressive molecular subtypes was observed in mBC compared to their paired primary tumors following the classification of Burstein and Lehmann [13, 15–17]. mTNBC were predominantly classified as BLIS (basal-like immune suppressive), mesenchymal or basal like-1.

## Immune checkpoint blockade for mTNBC: current status

Until recently, chemotherapy was the only therapeutic agent available for TNBC due to the lack of molecular targets. In contrast to early breast tumors, mTNBC rapidly becomes resistant to chemotherapeutic agents [7, 8, 18]. Immune checkpoints, among which the PD-1/PD-L1 axis, have shown to play an important role in T cell responses against tumor cells. Blocking immune checkpoints or its ligands enhances anti-tumor immunity and has tremendously changed the treatment of varies solid tumor types.

Initial trials evaluating immunotherapy for breast cancer focused on ICB-monotherapy and showed some clinical activity in mTNBC patients [19, 20]. These responses were more durable when compared to chemotherapy. Given the limited responses towards single-agent immunotherapy and the potential immunomodulatory properties of some chemotherapeutic agents, clinical trials subsequently combined immunotherapy with standard chemotherapy. The IMpasssion130-trial was the first phase 3 trial evaluating the efficacy of chemo-immunotherapy for mTNBC [21]. In this first-line trial, 902 patients with mTNBC were randomly assigned to receive nab-paclitaxel with either atezolizumab (anti-PD-L1) or placebo. Patients who had recurred after having a primary breast tumor were required to have a disease-free interval (DFI) of at least 12 months. Primary endpoints were progression-free survival (PFS) in the intention to treat population (ITT) and PD-L1 positive population, and hierarchical testing of the overall survival (OS) starting in the ITT-population followed by the PD-L1 population. The combination of atezolizumab with nab-paclitaxel resulted in significant improvement of PFS in both ITT- and PD-L1 positive population. These results did not translate into an OS-benefit in the ITT-population, but a seven-month improvement in OS was observed in the PD-L1 positive population (HR 0.71, 0.54–0.94), although not formally statistically tested [22].

Subsequently, additional evidence for the efficacy of a combined chemo-immunotherapy schedule was seen in the KEYNOTE-355 trial. Patients with mTNBC were randomly assigned to chemotherapy of investigator’s choice combined with pembrolizumab (anti-PD1) or placebo as first-line treatment [23]. Patients had a DFI of at least 6 months since completion of curative chemotherapy. Primary endpoints were PFS and OS with hierarchical testing in the PD-L1 CPS ≥ 10 population, PD-L1 CPS ≥ 1 population and ITT-population. Both PFS and OS were significantly improved with the addition of anti-PD1 therapy with a 7 months OS benefit (HR 0.73, 95% CI, 0.55–0.95, *p* = 0.019) in the PD-L1 CPS ≥ 10 population, but not in the PD-L1 CPS ≥ 1 population and therefore not formally tested in the ITT-population [24]. The improvement in PFS resulted in accelerated FDA approval for patients with PD-L1-positive CPS ≥ 10 mTNBC.

In contrast to these positive outcomes, the combination of paclitaxel with anti-PD1 in IMpassion131 failed to significantly improve PFS for previously untreated mTNBC. Although several possible explanations are proposed for the observed differences between the trials, cross-trial comparison does not allow firm conclusions [25]. Despite accelerated FDA-approval for atezolizumab with nab-paclitaxel for patients with PD-L1 positive mTNBC, Roche voluntarily withdraw the approval in August 2021 after the IMpassion131 failed to confirm these findings [26].

The addition of pembrolizumab to neoadjuvant chemotherapy (NAC) for patients with high-risk early-stage TNBC improved pCR rate by 7.5% (95% CI, 1.6–13.4) and resulted in an improved event-free survival (EFS) from 79.4% to 86.6% (HR 0.65, 95% CI, 0.48–0.88, *P* = 0.0025) in the KEYNOTE-522 trial [27]. Both neoadjuvant atezolizumab and durvalumab to NAC improved pCR rates in IMpassion-031 and GeparNuevo-trial respectively [28, 29]. The approval of neoadjuvant ICB has also implications for the therapeutic strategies for mTNBC since in the future patients diagnosed with mTNBC might have been treated with ICB before. Currently, no data is yet available how to treat patients with a recurrence after receiving prior ICB for their primary disease.

## Finding the optimal chemotherapy backbone for ICB

### Conventional chemotherapy as ICB backbone

Although the combination of chemo-immunotherapy is thought to have synergistic effects, the optimal chemotherapy backbone is still under debate. Initially, chemotherapy was considered to be immunosuppressive due to the cytotoxic effects on highly proliferating cells, thereby inducting myelosuppression [30]. On the contrary, preclinical data showed immunomodulatory properties of chemotherapeutics capable of inducing a more favorable TME with the exact effects varying across different classes of chemotherapy [31]. Anthracyclines, inhibitors of topoisomerase class II, can stimulate immunogenic cell death, whereas the platinum derivate cisplatin may promote upregulation of MHC class I expression and deplete immunosuppressive cells [32, 33]. In the KEYNOTE-355, pembrolizumab was added to different chemotherapy backbones either paclitaxel, nab-paclitaxel or gemcitabine-carboplatin. Since the chemotherapy backbone was determined by physician’s choice rather than randomization and variation in pretreatments across the cohorts was observed, the exact contribution of chemotherapy backbone and prior treatment on ICB-efficacy could not be fully elucidated [23]. These results indicate that the effects of chemotherapeutics on enhancing ICB efficacy warrants further investigation in future trials.

The TME and systemic immune response might also be influenced by dosing and sequencing of chemotherapeutic agents. Results from the phase II TONIC trial suggested that a short induction period of 2 weeks with cisplatin or doxorubicin, prior to ICB treatment could induce a more inflamed TME and enhanced ICB response in mTNBC patients [11]. These results support the idea that some patients could benefit from a short induction period instead of a concurrent chemo-immunotherapy approach to enhance ICB response thereby reducing chemotherapy-associated toxicities. Further research should explore which patients will benefit from either ICB-monotherapy, a concurrent approach or induction approach.

### Antibody drug conjugates in relation to immunotherapy

Recently, improvement in PFS and OS have been observed after treating patients with mBC with antibody-drug conjugates (ADC), which consists of an antibody conjugated with a biologically active cytotoxic compound [34]. The phase III ASCENT trial studied the efficacy of sacituzumab govetican (SG) compared to single agent chemotherapy for heavily pre-treated patients with TNBC, and was the first ADC to receive FDA-approval [35]. SG targets trophoblast cell surface antigen-2 (TROP2) to deliver its cytotoxic payload SN-38, a topoisomerase inhibitor [35]. All patients in the ASCENT trial, for which TROP2-expression could be evaluated, expressed TROP2 to some extend [36]. 56% of the patients had high expression of TROP2 according to their histochemical (H-) scores, while 26% had medium and 18% low H-score. A significant improvement in both PFS (HR 0.41; 95% CI, 0.32–0.52; *P* < 0.001) and OS (HR 0.48; 95% CI, 0.38–0.59; *P* < 0.001) has been observed. ADCs have also shown to be successful for patients with low expression of HER2 in the phase III DESTINY-Breast04 trial [37]. HER2-low is defined as HER2-expression of 1+ or 2+ without gene amplification. Treatment with trastuzumab deruxtecan (T-DXd), consisting of the monoclonal antibody trastuzumab targeting HER2, conjugated with the topoisomerase I inhibitor exatecan derivative resulted in significant improvement of PFS (HR 0.50; 95% CI, 0.40–0.63; *P* < 0.001 from 5.1 up to 9.9 months compared to chemotherapy of physicians’ choice in the total study population. Median overall survival was 23.4 months among patients treated with T-DXd compared to 16.8 months among patients treated with chemotherapy of physicians’ choice (HR 0.64; 95% CI, 0.49–0.84; *p* = 0.001). For patients with TNBC, PFS significantly improved from 2.9 months up to 8.5 months (HR 0.46; 95% CI, 0.24–0.89). There were, however, only 58 patients with mTNBC included in the trial. T-DXd has a direct effect on the HER2-expressing cells as well as a strong bystander effect. Approximately 30% of TNBC is HER2-low and could potentially benefit from this treatment approach.

It is expected that ADCs will soon become an important pilar in the treatment regime of mTNBC. Internalization of the cytotoxic payload of the ADC induces immunogenic cell death, resulting in the release of danger-associated molecular patterns (DAMPs) [38]. Subsequently, maturation and activation of dendritic cells (DCs) is stimulated which will migrate to lymph nodes to activate naïve T cells. The addition of ICB could further stimulate the anti-tumor immune response by unleashing T cells. ADCs loaded with a topoisomerase I inhibitor, such as T-Dxd and SG, increase tumor-infiltrating DCs and CD8+ T cells, and stimulate the expression of PD-L1 and MHC class I upon tumor cells. However, (pre-)clinical evidence to support this concept is so far limited. A phase IB trial studying the combination of T-DXd with nivolumab for HER2+ MBC or patients with pretreated HER2-low mBC showed ORR of 59.4% and 37.5% respectively. The phase Ib basket BEGONIA-trial showed an ORR of 66.7% in patients with HER2-low mBC treated with the combination T-DXd and durvalumab (anti-PD-L1) as first-line treatment. Additionally, another basket in the BEGONIA trial investigated the ADC datopotamab deruxtecan, which is a TROP2-directed ADC. Upon combination with durvalumab as first-line treatment for patients with mTNBC, an ORR of 79% was observed [39]. The phase II Saci-IO trial (NCT04468061) will evaluate the efficacy of SG in combination with pembrolizumab for mTNBC with PD-L1 negative disease [40], while the ASCENT-04 trial (NCT05382286) will evaluate the efficacy of this combination for PD-L1 positive mTNBC [41]. Both trials are currently open for enrollment. As for now, clinical data in TNBC is awaited and translational research is warranted to further dissect the potential synergy between ADCs and ICBs.

## Novel immunomodulatory avenues

Responses towards ICB are low with response rate of approximately 5%, however, response rates increase upon selection for PD-L1 positivity to around 20%. Ongoing (pre)clinical trials aim to further optimize ICB efficacy and widen its application beyond PD-L1 positive breast tumors.

### Dual checkpoint inhibition

Dual checkpoint inhibition targets multiple parts of the cancer immunity cycle simultaneously and could therefore be a promising strategy for patients less likely to respond to chemo-immunotherapy [42]. Although dual checkpoint inhibition has shown to be effective in more immunogenic tumors, it often comes at a cost of higher toxicity. So far, only limited IO-IO combinations have been approved for the treatment of solid tumors including ipilimumab (anti-CTLA4) with nivolumab for patients with melanoma, mesothelioma, hepatocellular carcinoma, renal cell carcinoma, non-small cell lung cancer and colorectal cancer, and relatlimab (anti-LAG3) combined with nivolumab (anti-PD1) for untreated unresectable or metastatic melanoma [42, 43]. Clinical trials are currently ongoing to study the efficacy of dual checkpoint inhibition for TNBC.

The first dual IO-combination that was tested across multiple cancer types was anti-PD1 combined with antibodies against cytotoxic T lymphocyte associated protein-4 (CTLA4). CTLA4 negatively regulates T cells by inhibiting proliferation and activation [44]. Simultaneously blocking CTLA4 and PD-1 showed enhanced efficacy of ICB compared to either single agent alone in multiple preclinical TNBC models. The efficacy of ipilimumab and nivolumab was evaluated in the multicohort phase II DART trial showing response in 3/17 patients with metaplastic mBC, predominantly being triple negative [45]. The phase II NIMBUS trial for TMB-H HER2-negative MBC showed objective responses in 4/30 patients, of which 10 had TNBC [46]. In addition, 4 weeks of neoadjuvant nivo/ipi induced immune activation in 9/15 patients with early-stage TNBC in the BELLINI-trial [47].

Other initiatives include the development of next-generation anti-CTLA4 antibodies. Probodies are antibody prodrugs which unmask their anti-CTLA4 binding site after being proteolytically cleaved in the TME, thereby preventing binding of the antibody in normal tissue reducing systemic toxicity [48]. The non-fucosylated (NF) antibody has increased anti-tumor activity via depletion of Tregs due to high affinity for the Fcy-receptor [49]. The next-generation compound botensilimab acts in a similar manner as the NF antibodies, and has shown to induce clinical activity in heavily pretreated patients with MSS-CRC [50].

The novel inhibitory immune checkpoint lymphocyte activation gene-3 (LAG-3) has been shown to be co-expressed with PD-1 and together reflect an exhausted T cell phenotype [51]. Preclinical data showed strong synergy between PD-1 and LAG-3 inhibitory pathways against tumor antigens and blockade of these checkpoints improved anti-tumor CD8 T cells responses [51, 52]. The combination has shown to successfully improve PFS in previously untreated metastatic melanoma patients compared to anti-PD1 therapy alone [43]. The exact proportion of TILs co-expressing both PD-1 and LAG-3 is not yet established [53, 54]. Nevertheless, dual blockade of LAG-3 and PD-1 is currently being tested for advanced breast cancer (NCT03742349, NCT03005782). Results of I-SPY2 trial investigating anti-LAG-3 and anti-PD1 in early-stage high risk HER2-negative BC, presented at SABCS 2022, showed a predicted pCR of 60% in patients with HR- HER2- disease, and 37% in patients with HR+ HER2- disease [55]. Importantly, a relatively high rate of adrenal gland insufficiency (21%) was observed with the combination anti-LAG3/anti-PD1 which requires special attention in follow-up studies.

The next wave of antibodies against inhibitory checkpoint includes antibodies against TIM-3, TIGIT, and VISTA and the landscape is expected to expand even further [51, 56]. Unraveling potential synergic effects between these inhibitory pathways is essential to provide a rationale for novel combination strategies.

### Bispecific antibodies

Bispecific antibodies (bsAbs), antibody-based molecules with two unique epitope binding sites, are used in cancer therapy to bridge immune cells and cancer cells, bringing them in more close proximity [57, 58]. The most commonly used epitopes on effector cells are CD3 on T cells forming bispecific T cell engagers (BiTEs) or CD16 on NK cells forming bispecific killer cell engagers (BiKEs).

The vast majority of bsAbs for cancer therapy are BiTEs [59]. Upon binding of both epitopes, T cells become activated and their cytotoxic activity is re-directed to the tumor cells without TCR specificity, co-stimulatory signals or antigen presentation being necessary. Multiple potential tumor-associated antigens (TAAs) have been identified in breast cancer, including HER2, TROP2, CEACAM, and EGFR [59, 60]. Although the majority of trials towards bsAbs focus on HER2-positive breast cancer, preclinical studies in TNBC showed promising immunomodulatory effects of BiTEs targeting TROP-2 resulting in reduced tumor growth [61]. In addition to traditional CD3 targeting bsAbs, bsAbs targeting immune checkpoint PD-(L)1 in combination with CTLA-4, TIM-3 or LAG3, are now being tested for other solid tumors, especially to overcome primary resistance to ICB [57].

A challenge for successful application of bsAbs is the limited availability of effector T cells in the TME of cold tumors and the potential recruitment of T cells with immunosuppressive effects such as regulatory T cells [57]. Future research should focus on getting a more inflamed TME in cold tumor types using bsAbs, and whether more specific T cell subgroups can be targeted to enhance bsAbs efficacy.

### Immunocytokines

Cytokines are cell signaling molecules, capable of attracting and reactivate effector T cells and suppress inhibitory signals to create a more ‘hot’ TME [62]. It was thought that administration of exogenous cytokines could potentially induce anti-tumor immunity [63]. Administration of IL-2 for advanced renal cell carcinoma and melanoma, and IFN-α for leukemia and melanoma induced anti-tumor immune responses leading to FDA-approval [64], but toxicity of these cytokines is significant, making these drugs not very attractive.

Another promising strategy is harenessing the immunomodulatory properties of the IL-2-pathway while diminishing its inhibitory properties using IL-2 variants (IL-2v). IL-2 enhances ex vivo expansion of immune cells for adoptive cell therapy and drives terminal differentiation by inducing expression of co-inhibitory receptors. The IL-2v H9T promotes CD8 T cell expansion, without induction of terminal differentiation, and combined with aPD-1 resulted in the generation of a highly functional CD8 T cell subset [65, 66]. The compound bempegaldesleukin stimulates the IL-2 signaling pathways via CD122 thereby increasing TILs, T cell clonality and PD-1 expression. Clinical activity has been observed upon treatment with bempegaldesleukin and nivolumab for mTNBC, however development of bempegaldesleukin has been discontinued [67, 68]. The novel compound IRX-2, consisting of multiple cytokines, primes the TME by increasing TIL-levels, PD-L1 expression and lymphocyte activation in a phase Ib trial in early TNBC. A follow-up phase II study is currently ongoing (NCT04373031) [69, 70]. Another strategy to preserve IL-2 signaling, while stimulating Treg depletion is administration of anti-CD25 antibodies. This CD25-blocking-IL-2-sparing antibody stimulates effector T cell development while depleting Tregs [71].

### Adoptive cell therapies: CAR T cells & TIL therapy

Chimeric antigen receptor (CAR) T cells are developed to recognize a specific tumor-antigen, thereby re-directing T cells to kill malignant cells [72]. Despite successes in hematological malignancies, the efficacy with CAR T cells in solid tumors is so far disappointing [73]. The immunosuppressive TME hampers the activity of CAR T cells, therefore, dual administration of CAR T cells with ICB might combat the cold TME seen in TNBC. In vitro experiments showed increased cytokine production and cytotoxicity upon this combination approach, while enhanced tumor control was observed in vivo [74]. In addition, CAR T cells targeting antigens present on both tumor cells as well as immunosuppressive cells could modulate the TME towards a more favorable state [73]. Finally, next-generation CARs or T cells redirected for antigen-unrestricted cytokine-initiated killing (TRUCKs) combine re-directed T cell activity with the immunomodulatory capacities of cytokines [75]. These CARs are armored CAR T cells that release pro-inflammatory cytokines upon engagement thereby inducing a more inflamed TME. Additional research to improve the efficacy of CARs for mTNBC is required.

Another type of adoptive cell therapy (ACT) is TIL therapy in which a patients’ own T cells are expanded ex vivo upon IL-2 stimulation and transferred back to the patient after receiving lymphodepleting chemotherapy [76, 77]. TIL therapy was initially developed to treat melanoma patients, and has shown to improve PFS compared to anti-CTLA-4 in patients refractory to anti-PD1 [78]. The first success in breast cancer has been observed in a patient with ER-positive mBC treated with pembrolizumab plus TILs reactive against four types of mutant proteins combined with IL-2 [79]. A follow-up pilot in 42 patients with mBC showed tumor antigen (TA)-reactive TILs in 67% of the included patients. Finally, six patients were enrolled in a protocol to receive enriched neoantigen-specific TILs combined with ICB, of which three patients showed an objective response, however, responses might be attributed to pembrolizumab rather than TIL therapy [80]. The majority of patients initially screened for this trial was not eligible for TIL-treatment. Studies towards ACT-approaches using DCs and NK cells are ongoing as well as development of TCR-therapy in which T cells are genetically engineered to express TA-specific TCRs [77, 81].

### Oncolytic viruses & cancer vaccines

Oncolytic viruses are engineered to target multiple steps in the cancer-immunity cycle [82]. Oncolytic viruses induce immunogenic cell death which result in the release of neo-antigens and danger signals, causing activation of innate and adaptive immune responses [82].

Currently, talimogene laherparepvec (T-VEC), a herpes simplex virus-1 (HSV) engineered to express the cytokine gene granulocyte-macrophage colony-stimulating factor (GM-CSF), is the only oncolytic virus approved for cancer therapy [82, 83]. Durable objective responses upon treatment with T-VEC were observed in a phase III trial for advanced melanoma [84]. Responses were predominantly observed in the injected tumor sites, whereas distant lesions were less responsive to T-VEC. Oncolytic viruses induce expression of immune checkpoints PD-1 and CTLA4, thereby sensitizing the tumor for ICB, which have been confirmed in a preclinical TNBC model in the neoadjuvant setting [82, 85–87]. A phase I trial for patients with HER2-negative breast tumors with residual disease after NAC showed 2/10 objective responses after treatment with T-VEC and atezolizumab [88]. More data towards the efficacy and safety of this combination for breast cancer is awaited.

Cancer vaccines stimulate a patients’ anti-tumor immune response by administration of cancer antigens, yet only one cancer vaccine received FDA approval [89, 90]. Clinical benefit was observed upon treatment with trastuzumab and the cancer vaccine nelipepimut-S (NPS) after completion of standard treatment in the TNBC subgroup of a phase II trial for high-risk HER2-low breast cancer [91, 92]. Whereas traditional cancer vaccines were loaded with tumor-associated antigens, novel vaccines consist of patient-specific neoantigens, which are more likely to elicit an T cell induced anti-tumor immune response [93].

## Biomarkers

Biomarkers to predict response to ICB in mTNBC patients are currently lacking, but are essential for selecting patients that will most likely benefit from ICB-treatment. Moreover, the rapidly expanding combination strategies require biomarkers to match individual patients to their most promising treatment option.

Expression of PD-L1 is currently the only accepted biomarker to select mTNBC patients for ICB [94]. The approval of pembrolizumab for PD-L1-positive, advanced TNBC was accompanied by approval of the concurrent DAKO 22C3 diagnostic assay. Despite the ongoing debate as a result of different predictive performance of the various assays, almost all mTNBC patients are currently being tested for PD-L1 expression. It must be noted that a small subgroup of mTNBC patients despite being PD-L1 negative respond to ICB.

Potential emerging predictive biomarkers for immunotherapy include TILs and TMB. TILs are indicative of pre-existing immunity which is essential for ICB-efficacy. KEYNOTE-086 showed that patients with higher levels of TILs were more likely to benefit from ICB [11, 74]. TILs predicted ICB-benefit, but not chemotherapy-benefit in the KEYNOTE-119 for untreated mTNBC [95]. Biomarker analyses in the phase II FUTURE-C plus trial showed an impressive ORR of 81.3% in the CD8-positive population to the triple combination of chemotherapy, anti-PD1 and an angiogenesis inhibitor for advanced, pretreated TNBC [96]. However, spatial distribution of TILs and their functional state might be even more important [47, 97].

It was suggested that patients with high TMB have higher level of neo-antigens and therefore more likely to benefit from ICB [98]. Although multiple analyses have shown a positive association between high TMB status and response to ICB in mTNBC, other trials did not confirm this association [11, 99–101]. Lack of standardized methods to determine TMB and the use of different cut-offs to define high TMB impedes the application of TMB as a predictive biomarker [21, 101, 102] Identification of (novel) biomarkers is needed to be able to provide personal treatment approaches.

## Conclusions & future perspectives

The approval of pembrolizumab in combination with chemotherapy for PD-L1 positive mTNBC as well as the addition of pembrolizumab to NAC for early-stage high risk TNBC marks a paradigm shift in the treatment of this aggressive breast cancer subtype [24, 27]. No data is yet available how to treat patients with a recurrence after receiving prior (neoadjuvant) ICB. Translational data will provide crucial insights in immunological and molecular changes after ICB-treatment that might provide clues on how to treat beyond progression on ICB. A small subset of patients might benefit from rechallenging ICB, however, efficacy of re-introducing the same ICB regimen is expected to be limited [103].

Besides addition of ICB to the treatment landscape of TNBC, ADCs have shown promising results as monotherapy, and are now evaluated in combination with ICB. Concurrent ADC and ICB comes at a cost of high toxicity and results in an expensive treatment. Their potential synergistic mechanisms need to be unraveled as well as whether a sequential instead of a concurrent approach might be as effective while reducing expenses and risk of severe toxicity.

TNBC-treatment is no longer a one-size-fits-all approach. The growing landscape of novel agents and combination strategies being tested requires critical thinking to determine which treatment fits best to which patient rather than setting-up trials for all-comers. For patients unresponsive to anti-PD1, an expanding range of clinical trial options with novel immunomodulatory agents and ADCs is available. Identification of predictive biomarkers could guide treatment decisions to enable more personalized treatment approaches. An additional challenge comes from determining type of treatment in the control arm in phase III trials. The rapidly changing treatment landscape may cause the control treatment to be obsolete at time of finishing the trial.

Taken together, the rapidly developing treatment landscape for this difficult-to-treat breast cancer subtype is moving towards a more personalized treatment approach, taking into account tumor characteristics such as molecular subtypes, pathological- and clinical characteristics such as PD-L1 expression, level of TILs, and prior lines of treatment. Patients with a highly immunogenic tumor might benefit from IO-monotherapy or IO-chemotherapy strategies, patients with less immunogenic subtypes require a stronger immunomodulatory strategy, while for patients with immune-dessert tumors non-IO treatment options should be considered.
